# Developments and Trends of Immunization in India: A Narrative Review

**DOI:** 10.7759/cureus.66547

**Published:** 2024-08-09

**Authors:** Pratik P Tawde, Zahiruddin Quazi, Abhay Gaidhane, Sonali G Choudhari

**Affiliations:** 1 Department of Community Medicine, Jawaharlal Nehru Medical College, Datta Meghe Institute of Higher Education and Research, Wardha, IND

**Keywords:** universal immunization programme, vaccine hesitancy, vaccine coverage, trends in immunization, vaccination, immunization

## Abstract

Immunization is a critical component of public health, and it undergoes various trends and developments over time. Immunization is considered one of humanity’s most remarkable journeys. It has helped save countless lives and will help save more if the goals of the 2030 Immunization Agenda (IA2030) are achieved. India is home to one of the largest global immunization programs. Immunization trends refer to the patterns and changes in the development of vaccines, the use of vaccines, and vaccination programs within a nation. There have been various trends in vaccine development and immunization in India. Trends can be influenced by various factors, including vaccine coverage, new vaccines, vaccine hesitancy, advances in medical science, technological innovations, public-private partnerships, public health policies, and public awareness. India has the world's largest immunization program regarding the number of vaccinations delivered, the recipients, the geographic spread, and the human resources required. In this review, we give an overview of various trends in immunization in India dating from the ancient era. A search of the PubMed, Scopus, and Google Scholar databases from 1945 to February 2024 was done to conduct a narrative review. This review includes English-language publications. This narrative review was conducted to summarize the government of India’s actions and strategies for immunization and vaccine development and also to trace the trends in immunization in India. It covers various vaccination programs of the Indian government and measures that were made to fight vaccine hesitancy and to enhance vaccination coverage such as developing vaccination roadmaps, scheduling vaccinations, utilizing digital health technology, monitoring vaccinations, and developing creative techniques. India has the world's largest Universal Immunization Program, vaccinating around 29 million pregnant women and 26.5 million infants annually. These trends reflect India’s ongoing commitment to immunization as a component of public health policy and efforts to address existing and emerging health challenges.

## Introduction and background

Immunization is a global health and development success story and is one of humanity’s most remarkable journeys. It has helped save millions of lives and will help save over 50 million over the next 10 years if the Immunization Agenda 2030 (IA2030) is achieved. To increase health and well-being, the IA2030 envisions a society where everyone, at every age, fully benefits from vaccinations [1]. Immunization has eradicated smallpox which alone was responsible for around 300 million lives in the twentieth century; it has also helped in the long road to eradicating polio. The power of immunization was also seen during the COVID-19 pandemic [2].

Any nation's immunization program's ability to be successful depends in significant part on local realities and national policies, although international organizations like the World Health Organization (WHO), the United Nations Children's Fund (UNICEF), and others promote global immunization drives and policies. This is especially true for a populous, diversified, and developing nation like India [3].

India has seen a lot of trends in immunization since ancient times, the pre-independence era till date right from importing vaccines from other countries to the establishment of vaccine units, being independent in the development of vaccines, production of vaccines, introducing various immunization programs, modification in National immunization programs, vaccine coverage, vaccine hesitancy, vaccination drives, etc. To comprehend the country's vaccination efforts, this overview analyzes historical events and current developments in vaccine development and immunization patterns in India. A comprehensive review of immunization trends in India is essential for maintaining and improving public health, ensuring the success of immunization programs, and achieving long-term health goals. The focus is on broader events and briefly addresses operational aspects and their efforts of the immunization program in the country.

## Review

Trends in ancient India till the first recorded smallpox vaccination (1802)

In the year 1545 AD in Goa, 8000 children approximately died due to the smallpox epidemic. There were few occurrences of smallpox in other parts of India as well; hence, few historiographers and doctors have mentioned smallpox as the ‘Indian plague’. Before smallpox vaccination, inoculation which is ‘the process of injecting an infective agent in a healthy person, which leads to often mild disease and preventing that individual from future serious disease’ was practiced in the country. The smallpox vaccine made its debut in India in 1802 four years after Jenner’s discovery [4]. The public's interest in immunization remained minimal even after Indian Medical Services staff worked diligently to develop a smallpox vaccination program. This was because of certain reasons like the requirement to pay a fee, the notion that immunizations are a form of punishment, and the belief that sickness results from a goddess' wrath. By 1850, vaccination rates had begun to rise, but there were still obstacles to overcome, including post-vaccination fatalities, problems, ineffective vaccination attempts, and opposition from certain Hindus who claimed that the vaccinations were from cows [5].

Sanitary and vaccination divisions were responsible for promoting vaccination during the 19th century. Dispensaries were a store for vaccines and were also used as a source for vaccination. The Bombay vaccination system, which was mostly dependent on traveling/tourist vaccinators, was in use in 1827. Other Indian provinces adopted the Bombay vaccination system, whereby traveling vaccinators provided almost two-thirds of the vaccinations and the dispensary system handled the remaining third [4]. In 1892, India executed the Compulsory Vaccination Act to curb the smallpox outbreak and increase vaccination rates. A cholera outbreak occurred in certain regions of India. In 1893 Agra, Uttar Pradesh, Dr. Haffkine conducted cholera vaccine experiments and demonstrated the vaccine's effectiveness. Later in the year 1987, during the plague epidemic, Dr Haffkine also developed the plague vaccine at Grant Medical College which was the first vaccine developed in India [6].

Trends during the pre-independence era (1900-1947)

There were long effects on vaccination efforts in India due to a few events in the twentieth century such as the diversion of vaccine providers to contain cholera and plague outbreaks, which had long-lasting consequences on immunization programs in India. The government gave the influenza pandemic, which claimed the lives of around 17 million Indians, a top priority. Following the Government of India Act 1919, the provision of health services, including smallpox vaccination [5], was done. Typhoid vaccination trials were done between 1904 and 1908 by the British Army Medical Committee's Anti-Typhoid Committee in India [7]. India recorded the greatest number of smallpox cases, and during 1944 and 1945, vaccination rates were at their lowest. After World War II, fewer cases occurred as attention returned to the smallpox vaccination [6,8]. By the early twentieth century, only plague, cholera, typhoid, and smallpox vaccines were available in India [5].

Trends during post-independence India (1947)

After gaining independence in 1947, India saw the introduction of more advanced bacterial vaccination methods. Nearly 10 years had passed since their global debut when Indian institutions for research and development of vaccines (R and D) lost their standing in comparison to other vaccine technology development centers. Table 1 shows the year gap between the vaccine introduced in India and other countries. During this period, routine productions and service functions, limited funding, a lack of encouragement for research and technology development, and a lack of an interdisciplinary strategy were among the factors that continued to pose a menace to India's efforts to develop vaccines [9].

**Table 1:** The global launch of vaccine technologies in India and other countries Compiled by author Dr. Pratik P. Tawde Source: [3] DPT: Diphtheria, pertussis and tetanus; DT: diphtheria and tetanus; TT: tetanus toxoid; OPV: oral polio vaccine; IPV: inactivated polio vaccine

Vaccine	Year of Introduction	
	India	Other Countries
Smallpox	1898	1890
Plague	1897	1897
Cholera	1892	1892
Yellow fever	1965	1941
Typhoid	1920	1915
TT	1972	1963
DT, DPT, BCG	1978	1963
IPV	1984	1955
OPV	1967	1962
Measles	1989	1980s
Hepatitis B vaccine	1997	1980s

India recorded the highest number of smallpox cases worldwide during its independence, and tuberculosis was thought to be a leading source of illness. The Indian government agreed to start the Bacille Calmette-Guerin (BCG) immunization program on a small scale in May 1948 [10]. India started its first immunization campaign in August 1948, and in 1949 it expanded to include all schools in nearly all of its states. The leeway of BCG vaccination in India was supported by the International Tuberculosis Campaign (ITC). India joined the World Health Organization (WHO) and started adhering to its and United Nations Children's Fund (UNICEF) policies. Many new Indian institutions were founded between 1950 and 1970, with some assistance from foreign organizations. The support of ITC ended in June 1951 and further, the Indian authorities in collaboration with UNICEF conducted the BCG vaccination. Financial support was provided by UNICEF and WHO gave the technical advice [10,11]. In all Indian Union states, a widespread BCG vaccination program was conducted between 1955 and 1956 [12]. The BCG vaccination was included in the National Tuberculosis Control Programme (NTCP) when it was launched in 1962 [11].

The "Feasibility Study for TB Prevention Trial" was a large-scale vaccine efficacy experiment of BCG carried out in Chingelput, Tamil Nadu in 1968. After all patients were followed up on, the experiment concluded in 1987 that the BCG vaccination had no discernible effect on preventing pulmonary tuberculosis in adults. As a result, the BCG vaccine for India was changed, and under the Universal Immunization Programme (UIP), BCG was administered at the end of the first year following birth [13]. In 1958, a resolution to eradicate smallpox was passed by the World Health Assembly (WHA). After four years, India launched the National Smallpox Eradication Programme (NSEP) intending to vaccinate every person for the following three years [14]. However, the coverage remained inadequate even after five years, therefore the program was reorganized with an emphasis on quick containment drives, surveillance, and epidemic investigation [15]. In 1969, the rotary lancet was replaced by a new bifurcated needle technique and the old liquid vaccine was also replaced by a heat-stable, freeze-dried vaccine which was more potent. Further with intensified efforts ‘Operation Smallpox Zero’ was there in the country from 1975-77 [16]. In India, vaccine was manufactured in 19 units and 12 units in the public and private sectors respectively. Other than BCG and smallpox vaccines the other vaccines available and manufactured in India by 1977 were diphtheria, pertussis and tetanus (DPT), diphtheria and tetanus (DT), tetanus toxoid (TT), oral polio vaccine (OPV). Measles vaccine was not yet manufactured in India [5]. Table 2 summarizes major historical milestones in India from ancient times till the present [5,17-20].

**Table 2:** Major historical milestones of vaccine development and immunization in India Compiled by author Dr. Pratik P. Tawde Source: [5,17–20]

Major Historical Milestones of Vaccine Development and Immunization in India	
Year	Immunization milestones
1600	Inoculation was practiced in India
1802	1^st^ documented smallpox vaccination was done
1892	Compulsory Vaccination Act passed by the Indian government
1896	Epidemic Act was passed
1897	1^st^ plague vaccine developed by Dr Haffkine in Bombay (now Mumbai)
1899	A laboratory for plague set up in Bombay (later named Haffkine Institute)
1910-1930	Many new vaccine institutes set up across India
1948	1^st^ BCG laboratory set up in Guindy, Madras (now Chennai)
1951	Mass campaigns of BCG started in India
1962	Launch of National Smallpox Eradication Programme The National Tuberculosis Control Programme initiated with the BCG vaccine
1970	Oral Polio Vaccine Trivalent (Sabin) was both developed and produced for 1^st^ time in India
1975	The last case of smallpox was reported
1977	The National Polio Surveillance Project was set up in collaboration of WHO and Govt. of India
1977	India declared smallpox-free
1978	Expanded Program of Immunization (EPI)
1985	Universal Immunization Program (UIP)
1985	AEFI surveillance system introduced with guidelines
1986	Technology Mission on Immunization
1992	Child Survival and Safe Motherhood (CSSM)
1997	The first recombinant DNA hepatitis B vaccine was developed in India.
2005	National Rural Health Mission launched.
2009	Pandemic flu (Novel H1NI:2009) vaccine developed by India.
2011	The last case of wild poliovirus was reported in Howrah, West Bengal.
2012	WHO declared India a Polio-free endemic country 2012 was declared as “Year of Intensification of Routine Immunization”.
2014	WHO certified India as a “polio-free country” along with the South East Asia Region (SEAR) countries of WHO. Mission Indradhanush Launched.
2015	The Inactivated Polio vaccine was introduced in UIP as a part of the Global Polio end-game strategy. Electronic Vaccine Intelligence Network (eVIN) was rolled out.
2016	Maternal and neonatal tetanus were eliminated.
2017	Intensified Mission Indradhanush Launched. Measles & Rubella (MR), Pneumococcal Conjugate Vaccine (PCV)& Adult Japanese Encephalitis (JE) were introduced in the UIP.
2019	Intensified Mission Indradhanush 2.0 was launched.
2020	Intensified Mission Indradhanush 3.0 was launched.
2021	The COVID-19 vaccination program along with the CoWIN portal was launched.
2022	Intensified Mission Indradhanush 4.0 was launched. Administered 200 crore COVID-19 Vaccine Doses.
2023	Launch of UWIN (Pilot Phase in 65 districts)

Expanded program on immunization (1977) onwards 

In 1974, the WHO launched the Expanded Programme on Immunization (EPI). In 1978 after India was declared smallpox-free, it started with a National Immunization program called the EPI. India is one of the largest countries in the world to start such a program. BCG, OPV, DPT, and typhoid-paratyphoid vaccines were introduced in EPI with infancy coverage of at least 80 percent [5,21,22]. The typhoid-paratyphoid vaccination was eliminated in 1981, and the TT vaccine for expectant mothers was introduced to the EPI in 1983 [5]. On November 19, 1985, the EPI was rebranded as the UIP. The regimen also included the vaccination against measles. Given that vaccination was one of the 20 points of the Prime Minister's program, it was more crucial. It was also named in the five National Technology Missions that were established in 1986 to achieve vaccine production and coverage self-sustainability [5]. In 1990 the target population of UIP included all pregnant women and children, and 85 percent of infants against vaccine-preventable diseases (VPD) which was further increased to cover 100 percent of infants by the year 1991. In 1991, the states and union territories assumed full responsibility for maintaining the cold chain, which was previously under the control of UNICEF and private organizations [23].

 *Polio Eradication*

Just as efforts to eradicate smallpox, there were similar efforts to control and eradicate poliomyelitis in India after the resolution was passed by the World Health Assembly in 1998 to eradicate polio by 2000. During the first two National Immunization Days (NIDs), 87 million children under the age of three received vaccinations. By 1997 children up to five years were targeted and about 125 million were vaccinated. There was a collaboration between The WHO and the Government of India to develop the National Polio Surveillance Project (NPSP) in 1997. The last polio case in India was reported in 2011 in a two-year-old girl, Rukhsar Khatun in Howrah, West Bengal. As of February 25, 2012, India was removed from the list of nations where polio was endemic [24]. Intending to reduce the VPD, the immunization program started in India and completed 30 years in 2008. No new vaccine was added to the UIP for 16 years since 1985 [5]. The Hepatitis B vaccine was introduced in a few states as a pilot in UIP during the year 2002/2003. After seven years in 2011, it became the seventh vaccine to be added to the UIP. The Haemophilus influenza type b (Hib) vaccine was made available in certain Tamil Nadu and Kerala states in 2011 [25]. India was the last nation to start a second dose of measles in the National Immunization Programme 2010 [5].

There were launches of programs such as Border Districts Cluster Strategy (BDCS), Urban Measles Campaigns, Immunization Strengthening Project (ISP), etc. to improve the coverage [26].

There were certain developments in India during 2010 such as one of the aims of immunization programs was to attain the target anticipated in the National Population Policy and National Health Policy of India. India launched its first multiyear strategic plan (MYP) in 2005. From 2012 to 2017 a new comprehensive MYP was also drafted [27]. Glass syringes were replaced by new types of syringes(auto-disable) only for UIP from 2005/2006. The National Technical Advisory Group on Immunization (NTAGI) was re-established in 2010 and was established in 2001. Adverse event following immunization (AEFI) guidelines were revised in 2005/2006 which were part of Immunization since 1985. To improve post-marketing surveillance for vaccines, the country initiated the National Pharmacovigilance Programme in July 2010 [28]. To monitor the potency of vaccines, Vaccine Vial Monitor (VVM) policy was adopted in UIP. India and other Southeast Asian nations committed to eradicating measles and controlling rubella and congenital rubella syndrome (CRS) by 2023. In 2008, all but India of the 193 Member States of WHO included two doses of measles vaccine in their national immunization programs. The second dose of Measles was introduced in 2010. India issued its first national policy in 2011. 2012-2013 was the Year of Intensification of Routine Immunization (IRI).

Immunization coverage was very slowly progressing at the rate of 1 percent per year from 2009 to 2013 [19]. Numerous improvements and developments were made concerning routine vaccination, vaccination campaigns (SIAs) against polio, measles, and Japanese encephalitis, monitoring adverse events following vaccination (AEFI), vaccine and cold chain logistics, vaccination program training, and strategic communication. Programs such as Border Districts Cluster Strategy (BDCS), Urban Measles Campaigns, Immunization Strengthening Project (ISP), etc. were launched to improve vaccine coverage [26]. To reach 90% vaccination coverage even faster Launched in 2014, the goal of Mission Indradhanush (MI) was to lower child mortality by vaccinating all children under the age of two and pregnant women with all accessible vaccinations [29].

 *Mission Indradhanush*

The Ministry of Health and Family Welfare (MoHFW) of the Government of India's main program is MI. It was initiated to immunize infants who had not had vaccinations or who had received only partial vaccinations against seven vector-borne diseases: whooping cough, tetanus, polio, TB, measles, and hepatitis B. Subsequently, more five diseases were added to the list of preventable illnesses: measles-rubella (MR), rotavirus vaccine, adult JE vaccine, inactivated Polio vaccine (IPV), adult JE vaccine, meningitis, and pneumonia (Hemophilus influenza type B infections). These illnesses were only present in areas where JE was endemic [30]. MI completed covering 701 districts in eleven phases increasing immunization coverage by 6.7% in one year [19].

Intensified Mission Indradhanush (IMI): Motivated by MI triumph and to fortify it even more Intensified MI was launched on 8th October 2017 to achieve 90% immunization in areas with constant low levels of coverage. The government of India initiated a Measles-Rubella (MR) campaign all over the nation including every child aged 9 months to under 15 years in a phase-wise manner. It covered a total of 121 districts across 24 states. It made an effort to change immunization practices from routine to Jan Andholan or people's movement. Compared to the National Family Health Survey- 4 (NFHS-4), there was an average increase in full vaccination coverage of 18.5% [30,31].

Intensified Mission Indradhanush (IMI) 2.0: Further 'IMI 2.0' was introduced by the Indian government to enhance the coverage of all accessible vaccines. 380 districts were focused on full immunization coverage with improved immunization sessions having flexible timings and mobile sessions. The mission was enhanced with political, administrative, and financial commitment [32]. IMI resulted in the vaccination of 7.41 lakh moms and 37.09 lakh children.

COVID-19

India is a leading nation for COVID-19 vaccine manufacture, administration, and clinical studies. India reported the first confirmed case of the virus on January 27, 2020. The Task Force was founded in April 2020 to advance internal research and development of COVID-19 medications, diagnostics, and vaccinations. By 2022, over 530,000 COVID-19 deaths and over 44.7 million cases have been documented in India [33]. To expedite the development of vaccines and to vaccinate the Indian populace, the Indian government declared that it would fund COVID-19 vaccine development with $120 million, naming the mission as COVID Suraksha. Using the Covaxin and Covishield vaccines, India launched its largest vaccination program on January 2021. Its goal was to immunize 3 billion people nationwide, representing all ethnicities. Only 30 million healthcare professionals received the COVID-19 vaccine at first. By December 2021, more than 1.45 billion vaccination doses had been given [34]. The COVID-19 vaccination program phase-wise is summarized in Table 3.

**Table 3:** COVID-19 phase-wise vaccination program Compiled by author Dr. Pratik P. Tawde from [20] HCWs: Healthcare workers; FLWs: frontline health workers

Phase	Date	Features
Phase 1	January16-February 28,2021	Vaccination was available only to HCWs and FLWs.
Phase 2a	March 1-31 March,2021	Vaccination was available only to persons above 60 years and 45 years with comorbidity.
Phase 2b	April 1 to 30	Vaccination was available to all above 45 age
Phase 3	May 1, 2021	Vaccination was allowed for 18 and above age.
Phase 4	January 3, 2022	Covaxin was available to children between 15-18 age group.
Phase 5	January 3, 2022	Precautionary dose available to HCWs, FLWs, and 60 plus age group
Phase 6	March 16, 2022	Precautionary dose to all individuals. Corbevax vaccine for children aged 12-14 years.

The Government of India has set up 2,34,772 COVID-19 vaccination centers all across the nation. 2,15,927 out of total CVCs being government-run and 18,845 privates. Each CVC was a team of five professionals including a doctor, two auxiliary nurses, one data entry operator, and one multi-purpose worker. The GOI provided free COVID-19 vaccination to all citizens [20]. The Arogya Setu app and CoWIN were used for the COVID-19 immunization registration process. Co-WIN is an all-inclusive, digital platform that runs on the cloud and is designed to facilitate the planning, execution, registration, and tracking of COVID-19 immunization at all levels, from the central to the peripheral. India is a developer and manufacturer of many COVID-19 vaccines [35].

Special Vaccination Drives for COVID-19

To thoroughly vaccinate those who were eligible for their first dosage and those who were due for their second dose, a door-to-door campaign known as Har Ghar Dastak was started in November 2021 which was running for a month and was organized as part of the Jan Bhagidari Andolan [36]. India started the Vaccine Maitri initiative. The initiative was to supply COVID-19 vaccines to other in-need countries. The initial beneficiaries were Bhutan and the Maldives [37]. Therefore, drawing from India's experience with its largest immunization campaign to date, over 1.8 billion doses of the COVID-19 vaccine were successfully administered as a consequence of coordinated efforts across the nation.

Intensified Mission Indradhanush (IMI) 3.0

To expand NIP's immunization coverage even further, IMI 3.0 was introduced on February 19, 2020. During the COVID-19 pandemic, children and pregnant women who had missed their vaccination shots were given extra attention. It was completed in two sessions, lasting fifteen days apiece in February and March of 2021. A total of 250 districts in 29 states were covered by IMI 3.0. About 2.24 lakh pregnant women and 9.58 lakh youngsters received vaccinations.

Intensified Mission Indradhanush (IMI) 4.0

In February 2022, the Indian government launched "IMI 4.0" to close the vaccination gap caused by the COVID-19 epidemic. It was carried out in 416 districts, including those where Azadi ka Amrit Mahotsav is recognized. From February 2022 to May 2022, three rounds of immunization programs were carried out. IMI 4.0 immunized 15.31 lakh expectant mothers and 59.66 lakh children.

Intensified Mission Indradhanush (IMI) 5.0

The Union Ministry of Health and Family Welfare inaugurated this year's main regular immunization program. Three rounds of the campaign were conducted, with a focus on vaccination coverage for rubella and measles. This campaign is special since it was run for children up to five years old in every district in the nation. IMI 5.0 employed U-WIN. More than 34 lakh children and 6 lakh pregnant women received vaccinations up until the second round of IMI 5.0.IMI 5.0 was promoted by many Social Media Influencers by appealing public to get vaccinated even if they had missed any dose at the nearest vaccination centers. There was also active participation from Jan Pratinidhis [18].

Introduction of New Vaccines in UIP

IPV was introduced to lessen the menace of switching from trivalent OPV to bivalent OPV and as a component of the global end-game plan for polio. The first IPV was released in November 2015. The Rotavirus vaccine intended to reduce the mortality and morbidity caused by Rotavirus diarrhea RVV and hence was introduced to the UIP in March 2016. Showing commitment towards the SEAR goal of Measles and Rubella elimination, the measles-rubella (MR) vaccine was launched in 2017. The coverage of the MR vaccine till September 2022 was 98.08%. Pneumococcal Conjugate Vaccine (PCV) was introduced to lower the morbidity and infant death rates caused by pneumococcal pneumonia. PCV was launched in a phased manner in UIP. The first phase was started in 2020-21 only in five states, by the beginning of the year 2022 it expanded nationwide. Tetanus and adult Diphtheria (Td) vaccine was introduced in adults after they began to contract diphtheria at a higher rate than school-age children. Therefore, starting in February 2019, pregnant women and children between the ages of 10 and 16 are administered the Td vaccination instead of the TT vaccine.

Success of MI

Although the program expanded over time, the NFHS indicates that only 44% of children were fully immunized in 1992-1993 and 62% in 2015-2016, respectively, indicating a modest but steady development towards full vaccination coverage (FIC). Further with more partnerships between sectors, planning, monitoring, and review more progress was seen.

Table 4 summarizes the full immunization coverage in NFHS and the Integrated Child Health & Immunization Survey-(INCHIS) [27].

**Table 4:** Full immunization coverage in National Family Health Surveys (NFHS) and Integrated Child Health & Immunization Survey (INCHIS) Compiled by Author Dr. Pratik P. Tawde Source: [27]

Sr.No	Source	Full Immunization Coverage(%)		
		Urban	Rural	Total
1.	National Family Health Survey-1	-	-	-
2.	National Family Health Survey-2	51.9	29.3	34.5
3.	National Family Health Survey-3 (2005-06)	57.6	38.6	43.5
4.	Coverage Evaluation Survey (2009)	67.1	58.5	61.0
5.	National Family Health Survey-4 (2017-18)	63.9	61.3	62.0
6.	National Family Health Survey-5 (2018-19)	75.5	76.8	76.4
7.	Integrated Child Health & Immunization Survey-INCHIS (2016)	75.9	68.9	70.8
8.	Health Management Information System HMIS (2017-18)	-	-	86.7
9.	Health Management Information System HMIS (2018-19)	-	-	87.04
10.	Health Management Information System HMIS (2019-20)	-	-	92.83
11.	Health Management Information System HMIS (2020-21)	-	-	87.8

Vaccine coverage

**Figure 1:**
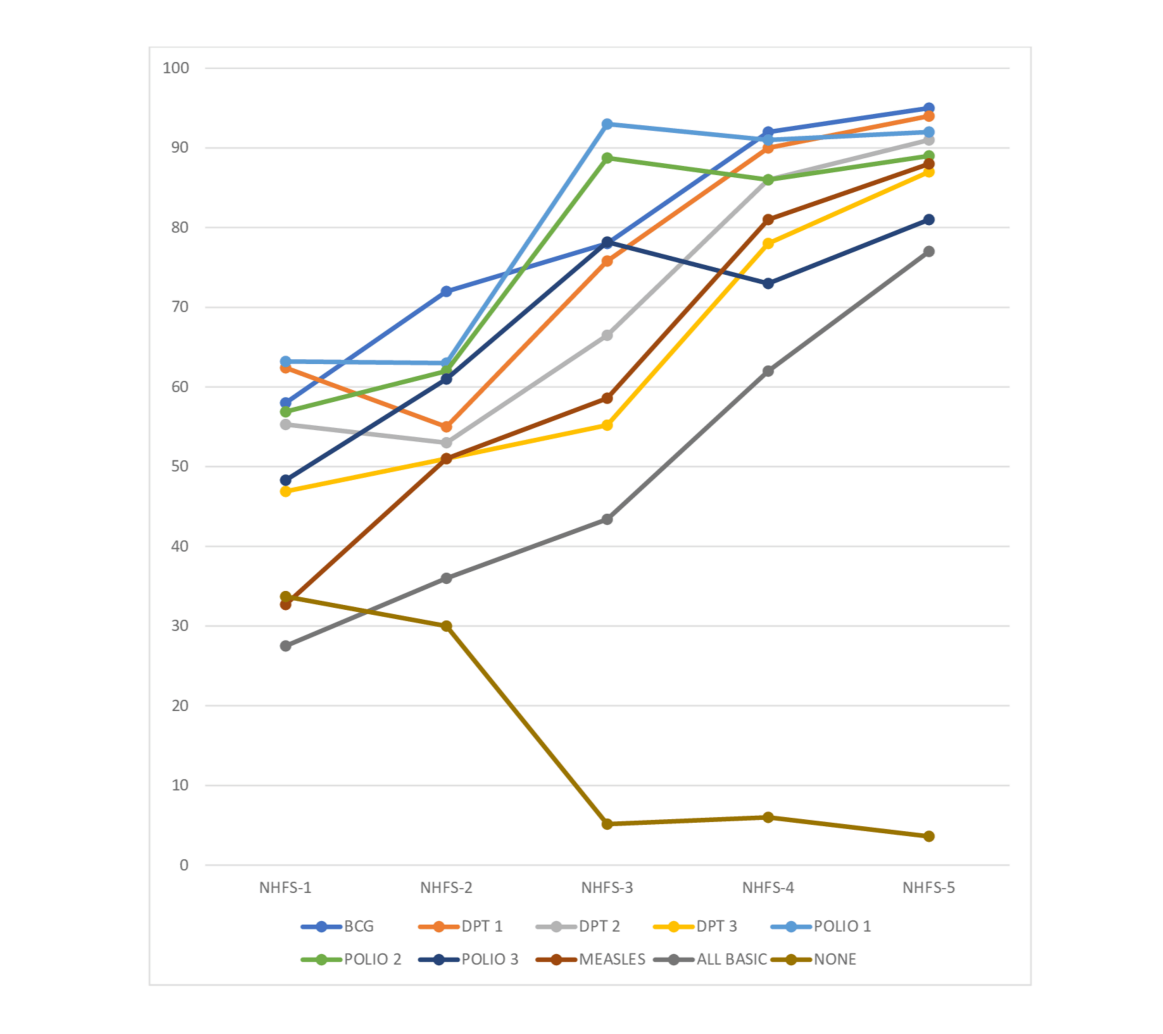
Trend of vaccine coverage over a period of time Compiled by author Dr. Pratik P. Tawde Source: [38]

Vaccine coverage in India has improved drastically in the last two decades. The progress was stagnant till 2013 hence MI was launched to boost vaccine coverage of the entire country in phases. The trend of vaccine coverage of BCG, DPT, Polio, other basic vaccines, and those who haven’t received any vaccine at all according to the NFHS is given in Figure 1 [38].

Vaccine confidence and hesitancy

For many years, vaccinations have helped to lower the morbidity and mortality of VPDs. Despite the success of immunization low vaccine confidence, vaccine hesitancy, vaccine delay, and refusal administration of vaccines have been seen in the nation. Vaccine hesitancy is a “delay in acceptance or refusal of vaccines despite availability of vaccine services” as defined by the WHO. Over the past 20 years factors that have been influencing the above are traditional, religious, and cultural practices, localized pockets of hesitancy, lower education status, lack of awareness of the time and place of vaccination, concerns and rumors about vaccine adverse effects, and safety and vaccine shortage [39].

There are generally three types of factors related to vaccine hesitancy: (i) Vaccine specific: there is concern about cost, safety, and adverse effects of the vaccine; (ii) Individual: There are certain doubts about health benefits and, an absence of confidence in the healthcare system and vaccination programs; (iii) Contextual which comprises socio-economic level, education, religion, and traditions. A few examples of vaccine hesitancy seen in India are stated below. 

The polio vaccine campaigns were affected after some communities believed that there was pig blood present in the vaccines and that the vaccine was related to sterility, later this disbelief was cleared by religious leaders along with community influencers which further led to the successful elimination of poliomyelitis [40,41]. There were safety concerns regarding the human papillomavirus (HPV) vaccine after the deaths of seven girls during the trial in 2010. It was cleared after the inquiry report that the reason for deaths was not HPV vaccines [42]. A recent example of vaccine hesitancy is COVID-19. In the early phases of the vaccine effort, there have been reports of population opposition and anxiety around COVID-19 vaccination. The vaccine hesitancy rate among the Indian population was 23 percent, and the two reasons were vaccine confidence and delay of vaccination [43].

Adult vaccines

Adults are typically affected by VPDs during outbreaks that cause high rates of morbidity, death, and financial burden on the nation [44]. Adults are more likely to have VPD outbreaks due to the following key causes: (i) adult immunization is not available; (ii) immunity is diminishing; (iii) age-related factors; and (iv) epidemiological shift [45]. Unvaccinated adults can also be at risk of spreading diseases to unimmunized infants (e.g. pertussis). If adult vaccination coverage is improved it will reduce healthcare costs and directly be significant to individual families and communities [44,46,47]. Coverage of vaccination of adults in India is held to be less [48]. Vaccine guidelines have been published for all adults and specifically for women also by various associations and Indian societies, but there are no systematic programs to recommend, promote, fund, and nationally adopt these guidelines [45,49-52]. However, due to a lack of national norms and a lack of perception of necessity, adult immunization coverage in India is now quite low [48]. TT at the age of 16 years and pregnancy to protect newborns against tetanus is the only vaccine that is nationally recommended [18]. It is replaced by tetanus and diphtheria toxoid (Td) [53-55].

TECH/system strengthening of immunization

Several techniques and systems were introduced in India to boost, impact, and surveillance of immunization.

Technological Advancement in Vaccines

The advancement and effective use of mRNA vaccines, like those for COVID-19 from Pfizer-BioNTech and Moderna, signify a major technological achievement. mRNA vaccines like AstraZeneca were domestically produced and used in India as they can be quickly developed and are highly effective. Viral vector vaccines are also effective and have the advantage of storage. There are recent advances in Nanoparticle and Protein subunit vaccines that aim to improve vaccine stability and immune response. These vaccines had great impact in reducing COVID-19 cases, hospitalizations, and deaths. They also helped in COVID-19 vaccination campaigns thus demonstrating India's ability to mobilize resources rapidly and reach diverse populations, including remote areas.

Surveillance and Action for Events Following Vaccination (SAFEVAC)

It is a web gateway created to improve AEFI surveillance in India in May 2019. Previously, the AEFI cases were recorded manually now with the help of SAFEVAC they are digitally recorded, speeding up the process and minimizing the loss of data due to transfer from the District to the State/National level.

Surveillance for VPDs

It was initiated in 2015 with surveillance of Diphtheria, Pertussis, and Tetanus. Acute Flaccid Paralysis (AFP) surveillance for Polio and Fever and Rash surveillance for Measles and Rubella are functional in the entire nation.

Effective Vaccine Management (EVM)

It is a widely recognized instrument for a secure and efficient vaccination supply chain. India conducted its first nationwide EVM assessment in 2013. Five years later in 2018, second assessment was done which observed a National EVM score of 68%. Third National assessment was done in 2022. 

Electronic Vaccine Intelligence Network (eVIN)

The Indian government launched SMS-based (e-VIN) to provide real-time stock monitoring of vaccination, to strengthen the body of evidence supporting improved vaccine delivery, purchasing, and planning policies. The Indian-developed eVIN system digitizes vaccine supplies and uses a smartphone application to track the cold chain's temperature [56].

COWIN

The COWIN portal was created during COVID-19 to facilitate the organization, execution, oversight, and assessment of COVID-19 immunization programs in India.

Strategies and packages for information education and communication (IEC) are as follows: The "5 Saal 7 Baar initiative," which disseminates knowledge about AEFI, MCP cards, and immunization regimens, to raise vaccine confidence and raise understanding of the risks associated with not vaccinating a kid for which a framework for risk communication was created, routine Immunization FAQs were created to dispel misunderstandings around vaccination and offer thorough information about it, frontline staff members receive BRIDGE (Boosting Routine Immunization Demand Generation and Expansion) training to improve their interpersonal communication abilities, IEC packages for novel vaccinations, which comprise the creation of social media creatives, audio-visual spots, posters, banners, and booklets regarding particular vaccines.

Impact on Public Health

Technological advancements and targeted campaigns have increased immunization coverage, especially in rural and underserved areas. The introduction of new vaccines has broadened protection against various diseases. We have seen successful eradication of polio in 2014 and significant reductions in diseases like measles and tetanus highlight the effectiveness of India’s immunization programs. Real-time data collection and monitoring systems have improved disease surveillance and outbreak response, enabling timely interventions.

Thus, India’s immunization campaigns and technological advancements have had a significant positive impact on public health by improving vaccination coverage, reducing disease burden, and enhancing health system capabilities. Continued innovation, strategic planning, and addressing challenges like vaccine hesitancy and logistical issues will be essential for sustaining and furthering these gains.

The success

Nearly 26 million infants are vaccinated against 12 VPD and around 600 million doses are administered to children every year under UIP in India. India has been successfully running polio campaigns on a large scale for the past 20 years [18]. In India, vaccination rates against the measles-rubella vaccine have reached 90% after a triumphant vaccination drive that, between 2017 and 2020 vaccinated approximately 324 million children between the ages of 9 months and 15 years [57]. With more than 65 percent of the population fully vaccinated against COVID-19 (up from zero in January 2021), India has the greatest COVID-19 immunization coverage in the world. India also has a higher proportion than global COVID-19 vaccination coverage [58]. Given India's vast and diverse population, there are many practical challenges faced.

Geographical and Demographic Challenges

States like Arunachal Pradesh, Jammu & Kashmir, and North-Eastern states make transportation of vaccines and maintaining cold chain in these areas difficult due to terrains and poor infrastructure. Providing vaccines to every person in urban slums and high-density areas is difficult due to the limited access to healthcare facilities. The migrating population makes it difficult to track and ensure complete vaccination coverage.

Socio-Cultural Challenges

Myths, spreading false information, and cultural and religious beliefs about vaccination influence the attitude toward vaccination leading to resistance and fear among certain communities further adding up the vaccine hesitancy.

Healthcare System Challenges

Shortage of trained healthcare professionals and vaccinators, lack of storage facilities and equipment, and maintaining the temperature of vaccines from production to administration were certain challenges in vaccine coverage particularly in rural areas.

Logistical and Supply Chain Challenges

Immunization schedules were affected because of variability in vaccine supply, delays in production, and inefficient inventory management systems which was particularly seen during COVID-19 vaccination. 

Vaccination monitoring and planning

India is deploying vaccinations through a whole-government strategy that involves state governments, the country's 19 federal ministries, as well as other government agencies, and other apex government bodies like the National Institution for Transforming India and the Indian Council of Medical Research (NITI Aayog), because of this all the Statewide vaccination campaigns are rigorously supervised by government institutions like the PMO, Parliament and courts, which established the national COVID-19 National Expert Group on Vaccine Administration for COVID-19 (NEGVAC) and guided all aspects of the vaccination strategy, including delivery methods, prioritizing safe surveillance, and equitable vaccine procurement and distribution [59].

Implications of historical trends on current policies and future directions

The significance of immunization programs is highlighted by past achievements in controlling, managing, and eradicating infectious diseases throughout history. Current strategies prioritize compulsory vaccinations for school admission, public education initiatives, and actions to secure vaccine availability and cost-effectiveness. Vaccine hesitancy was difficult to manage earlier but now policies like transparent communication, engagement with community leaders, and leveraging social media to disseminate accurate information. International collaboration in immunization has always been a success in controlling diseases since history. The focus of current policies is on global partnerships to combat outbreaks and ensure vaccine equity, particularly in low- and middle-income countries.

Future directions may include the development of new vaccines using advanced technologies like mRNA and making universal vaccines. It is essential to make sure that health systems are strong and able to manage extensive immunization campaigns. This encompasses investing in healthcare facilities, educating healthcare professionals, and improving supply chain management. The recent COVID-19 pandemic provides historical insights for future preparedness plans. Strategies should focus on large vaccine production, hoarding necessary resources, and establishing systems for quick reactions to new infectious diseases. Disparities in vaccine access remain a major concern. Future policies will likely prioritize equitable distribution of vaccines, especially in underserved communities, both domestically and globally.

## Conclusions

India has the world's largest Universal Immunization Program, vaccinating around 29 million pregnant women and 26.5 million infants annually. India has learned from its past to attain the desired policy objectives and to provide the advantages of immunization to all eligible citizens. Since 2014, MI has been completed in 12 phases across the nation. As a result of ongoing efforts to improve routine immunization and sporadic intensification pushes conducted in previous years, MI has so far vaccinated more than 5.06 crore and 1.25 crore children and pregnant women respectively. Immunization has changed the game globally, helping to stop the spread of coronavirus infections and eradicate smallpox. India has been an active part of the development and trends and is currently in the most successful immunization period. This review article overviews various trends and developments in immunization in India. Future research and policy directions in immunization should focus on innovation in vaccine technology, understanding and addressing vaccine hesitancy, strengthening immunization infrastructure, and fostering global and regional collaborations. By prioritizing these areas, we can improve immunization coverage, enhance public health outcomes, and prepare for emerging infectious diseases.
